# Urticaria

**Published:** 1841-11

**Authors:** 


### URTICARIA.

In a late letter from a distant state, a medical friend says—"If you know of any means of cure for *chronic* urticaria, besides those usually laid down in the books, you will oblige me by stating them, either through the Journal or by letter. I have a patient who has had the disease from infancy, and been treated by the most skilful physicians in this part of the country—all to no effect. She is 19 years old, and apparently in good health in all her functions. The eruption makes its appearance more or less every day, and is very distressing. Can it be cured."

The demand of our friend is one which we cannot meet. We can only confess our own inability, and call upon our more skilful brethren to answer the question affirmatively and point out the means.

It has happened to us, to see many cases, both acute and chronic of this singular malady; the former have readily yielded—the latter for the most part have proved refractory. We have often observed a high degree of nervous irritation connected with *acute* urticaria, never a decided phlogistic diathesis, and we have found the administratisn of large doses of opium or its preparations, followed by an emetico-cathartic, of great efficacy. Bloodletting has not been of much benefit. We have, now and then, seen a compound of nitrate and bitartrate of potash do good. But in *chronic* urticaria, we have had little success. It has been said to depend on gastric or hepatic derangement, but we recollect the case of a gentleman, who labored under it for many months, during which his digestion was good, and the biliary secretion perfect, both in quality and quantity. He suffered most in winter; and the intolerable itching which arose under exposure to cold, was rèmoved by the heat of a warm fire. Years have passed away, during which his general health has been good, but he is still more or less affected with this malady in winter.

We shall be happy to publish any thing that is valuable on this subject. D.

